# Urologists’ Estimation of Online Support Group Utilization Behavior of Their Patients With Newly Diagnosed Nonmetastatic Prostate Cancer in Germany: Predefined Secondary Analysis of a Randomized Controlled Trial

**DOI:** 10.2196/56092

**Published:** 2025-04-07

**Authors:** Philipp Karschuck, Christer Groeben, Rainer Koch, Tanja Krones, Andreas Neisius, Sven von Ahn, Christian Peter Klopf, Steffen Weikert, Michael Siebels, Nicolas Haseke, Christian Weissflog, Martin Baunacke, Christian Thomas, Peter Liske, Georgi Tosev, Thomas Benusch, Martin Schostak, Joachim Stein, Philipp Spiegelhalder, Andreas Ihrig, Johannes Huber

**Affiliations:** 1 Department of Urology University Hospital Heidelberg Heidelberg Germany; 2 Department of Urology Medical Faculty Carl Gustav Carus Technical University Dresden Dresden Germany; 3 Department of Urology Philipps University of Marburg Marburg Germany; 4 University Hospital Zürich University of Zurich Institute of Biomedical Ethics and History of Medicine Zurich Switzerland; 5 Department of Urology Brüderkrankenhaus Trier Trier Germany; 6 Department of Urology Alexianer St. Hedwig-Krankenhaus Berlin Germany; 7 Urological Joint Practice Urologie Pasing Prof Dr med Michael Siebels & Dr med Nicolas Haseke Munich Germany; 8 Urological Joint Practice am Weißen Hirsch Dresden Germany; 9 Urological Practice Dr Gnann und Dr Liske Stuttgart Germany; 10 Urological Practice Mannheim Germany; 11 Urological Practice Neustadt/Saxony Germany; 12 Department of Urology, Uro-Oncology, Robot-Assisted and Focal Therapy University of Magdeburg Magdeburg Germany; 13 LOGICURO Berlin Germany; 14 Department of Urology KRH Klinikum Großburgwedel Burgwedel Germany; 15 Urological Joint Practice Urologie Neandertal Mettmann Germany; 16 Division of Psychooncology Department of General Internal Medicine and Psychosomatics University of Heidelberg Heidelberg Germany

**Keywords:** peer support, prostate cancer, online support, health services research, randomized controlled trial, decision aid

## Abstract

**Background:**

Due to its high incidence, prostate cancer (PC) imposes a burden on Western societies. Individualized treatment decision for nonmetastatic PC (eg, surgery, radiation, focal therapy, active surveillance, watchful waiting) is challenging. The range of options might make affected persons seek peer-to-peer counseling. Besides traditional face-to-face support groups (F2FGs), online support groups (OSGs) became important, especially during COVID-19.

**Objective:**

This study aims to investigate utilization behavior and physician advice concerning F2FGs and OSGs for patients with newly diagnosed PC. We hypothesized greater importance of OSGs to support treatment decisions. We assumed that this form of peer-to-peer support is underestimated by the treating physicians. We also considered the effects of the COVID-19 pandemic.

**Methods:**

This was a secondary analysis of data from a randomized controlled trial comparing an online decision aid versus a printed brochure for patients with nonmetastatic PC. We investigated 687 patients from 116 urological practices throughout Germany before primary treatment. Of these, 308 were included before and 379 during the COVID-19 pandemic. At the 1-year follow-up visit, patients filled an online questionnaire about their use of traditional or online self-help, including consultation behaviors or attitudes concerning initial treatment decisions. We measured secondary outcomes with validated questionnaires such as Distress Thermometer and the Patient Health Questionnaire-4 items to assess distress, anxiety, and depression. Physicians were asked in a paper-based questionnaire whether patients had accessed peer-to-peer support. Group comparisons were made using chi-square or McNemar tests for nominal variables and 2-sided *t* tests for ordinally scaled data.

**Results:**

Before COVID-19, 2.3% (7/308) of the patients attended an F2FG versus none thereafter. The frequency of OSG use did not change significantly: OSGs were used by 24.7% (76/308) and 23.5% (89/308) of the patients before and during COVID-19, respectively. OSG users had higher levels of anxiety and depression; 38% (46/121) reported OSG as helpful for decision-making. Although 4% (19/477) of OSG nonusers regretted treatment decisions, only 0.7% (1/153) of OSG users did (*P*=.03). More users than nonusers reported that OSGs were mentioned by physicians (*P*<.001). Patients and physicians agreed that F2FGs and OSGs were not mentioned in conversations or visited by patients. For 86% (6/7) of the patients, the physician was not aware of F2FG attendance. Physicians underestimated OSG usage by 2.6% (18/687) versus 24% (165/687) of actual use (*P*<.001).

**Conclusions:**

Physicians are more aware of F2FGs than OSGs. Before COVID-19, F2FGs played a minor role. One out of 4 patients used OSGs. One-third considered them helpful for treatment decision-making. OSG use rarely affects the final treatment decision. Urologists significantly underestimate OSG use by their patients. Peer-to-peer support is more likely to be received by patients with anxiety and depression. Comparative interventional trials are needed to recommend peer-to-peer interventions for suitable patients.

**Trial Registration:**

German Clinical Trials Register DRKS-ID DRKS00014627; https://drks.de/search/en/trial/DRKS00014627

## Introduction

### Background

Approximately 60,000 men in Germany are diagnosed with prostate cancer (PC) annually, and approximately 95% have nonmetastatic disease [1,2]. For these patients, deciding among surgery, radiation, and active surveillance represents a major challenge. Individual preferences strongly influence the decision-making process [3]. To clarify these convictions, exchange with other patients seems to be important. In addition to traditional face-to-face support groups (F2FGs), online support groups (OSGs) and digital health tools are becoming increasingly important [4-6]. Due to societal changes caused by the COVID-19 pandemic, OSGs have become even more relevant.

### Importance of Self-Help for Patients With PC

In a recent review of interviews, informational support, shared experiences, and learning from others were identified as key benefits of peer-to-peer support groups [7]. Both F2FGs and OSGs support information exchange and provide emotional support [8]. The German PC S3 guideline, which is an evidence- and consensus-based instrument for improving the early detection, diagnosis, and treatment of PC, recommends that patients “should be informed about the option of contacting a peer-to-peer support group” [1]. The PC guideline is published by the German Association of Scientific Medical Societies, the German Cancer Society, and the German Cancer Aid Foundation, under the auspices of the German Society of Urology. This guideline is intended to support men and doctors in deciding on early detection measures. The recommendations are aimed at all those affected and all professional groups of the participating specialist societies and organizations. The S3 guideline is valid until the next update (maximum 5 years; currently until May 2029). Statements/recommendations for which the guideline group has decided to work on the basis of expert consensus are labelled as expert consensus. In Germany, the Association of the Scientific Medical Societies in Germany coordinates the development of guidelines. It divides guidelines into 4 classes. The S3 guideline fulfils the best possible evidence: the commission is representative, and the knowledge is systematically collected and evaluated. Moreover, there is a regulated procedure for arriving at a standardized recommendation in the event of different assessments within the commission.

In a survey, one-third of the urologists stated that after the initial diagnosis of nonmetastatic PC, they would mention the possibility of contacting a peer-to-peer support group [9]. Our recent systematic literature review revealed the effects of different forms of OSGs [10]. In all the studies, OSGs played a major role in treatment decisions and in the social environment. Information exchange in the OSGs was the predominant feature, but emotional support also played an important role. Nevertheless, there is still a relevant knowledge gap concerning the reasons for participation in peer-to-peer PC support groups and their effects.

### Objectives

The aim of this study was to investigate the utilization behavior of patients and physician advice concerning F2FGs and OSGs for patients with newly diagnosed PC. We hypothesized the greater importance of OSGs to support treatment decisions. Moreover, we assumed that this form of peer-to-peer support was underestimated by the treating physicians.

## Methods

This was a predefined secondary analysis of a randomized controlled trial (RCT) comparing an online decision aid versus a printed brochure for patients with nonmetastatic PC [11].

### Online PC Decision Aid

We previously described the online PC decision aid “Entscheidungshilfe Prostatakrebs” in detail [12-14]. The browser-based web-based tool offers guideline-based content using 17 educational videos in the German language with a total duration of more than 1 hour. While using the decision aid, the patient can enter all relevant personal and medical information. This input is used to personalize the videos according to 3 strata (oncologic risk, life expectancy, and erectile function) and to create a 1-page summary as a basis for the following discussion. In contrast, the patient guideline “Prostate cancer - localized disease,” comprising over 100 pages, is only available in printed form or as a PDF file [15]. The brochure is also based on current medical knowledge and recommendations from the German S3 guideline on PC. The brochure contains a section with guiding questions for a summary, which the patients must prepare themselves.

### RCT: Evaluation of a Patient-Oriented Decision Aid and the German Health Care Situation for Nonmetastatic PC

The RCT study size was calculated for the primary outcome, that is, the treatment decision after 14 months considered as a binary variable (deferred treatment vs other). We assumed a 14% deferred treatment rate in the control group, a detectable minimum difference of 7%, and a 14-month dropout of 23%. Due to the separate analyses for patients with lower and higher oncological risk, both subgroup analyses had to be α-adjusted. This procedure resulted in the analysis of 462 patients per group for a 1-sided chi-square test with power of 80% and α=.05/2. Therefore, we planned to randomize 1200 patients. As this calculation was sufficient for a simple group comparison, we also considered the comparison of the secondary analysis to be meaningful.

Patient recruitment occurred at 116 urological practices and clinics throughout Germany. The inclusion criteria were age between 18 and 80 years, histologically confirmed adenocarcinoma of the prostate, no clinical evidence of metastases (cM0 or cMx), prostate-specific antigen <100 ng/ml, and no primary treatment. In addition, the patient had to have internet access and an email address. After the initial diagnosis of PC was confirmed, the treating physician offered the patient the opportunity to participate. For both physicians and patients, the study design included 2 surveys: at T0 after the initial diagnosis and at T1 in the follow-up of 1 year. The study procedure was integrated into routine care: patients in whom the tissue sample confirmed the suspected diagnosis of PC and who fulfilled the inclusion criteria were informed by the study doctor about participation in the study. If consent was given, patients were randomized at T0 to the intervention group (use of the online PC decision aid) or the control group (use of the printed PC patient guideline and questionnaire). The intervention group completed the questionnaires online after using the PC decision aid. The study physicians and the patients in the control group completed the paper-based documentation. In order to remind the study participants in the intervention group of the repeat survey at T1, they received a reminder by email. Patients in the control group were sent the follow-up documentation forms by post. If the completed questionnaires were not received, a postal reminder was sent. Clinical data were separated from personal information by a data trustee.

The study physicians faxed the completed and signed patient enrollment forms to the study office. The form includes the enrollment date, the assigned study arm, and basic clinical data, but no personal data. The physicians received the basic physician documentation (T0) at 2-4 weeks after patient enrollment at the representation and not at patient enrollment and a “physician final documentation” after 1 year (T1).

In the RCT, there were no differences between the intervention and control groups in terms of treatment decisions, knowledge, acceptance, decision conflict, physician-patient communication, anxiety and depression, decision regret, or quality of life [11].

### Secondary Analysis: Peer-To-Peer Counseling

For this secondary analysis, we evaluated data from patients and their treating physicians concerning a wide range of aspects of peer-to-peer counseling. Therefore, in addition to several sets of anamnestic and medical data, patients and physicians were asked independently at T0 whether F2FGs and OSGs were involved in the physician-patient consultation. We asked patients whether they had used traditional self-help (F2FG) or online self-help (OSG) after being diagnosed with PC. Additionally, we used the validated Distress Thermometer and the Patient Health Questionnaire-4 items to assess distress, anxiety, and depression at both T0 and T1 [16,17]. At T1, patients were asked whether they had visited F2FGs and OSGs, including consultation behaviors. We also questioned whether they had ever attended a meeting of a PC self-help group and, if so, how often they had attended. Additionally, they were asked about their attitudes concerning initial treatment decisions. Physicians were asked whether their patients had accessed peer-to-peer support services. For this purpose, we applied previously established questionnaire items from our working group [5]. The topic-specific questionnaires are provided in Multimedia Appendix 1.

Since the survey period extended from August 2018 to October 2021, the effects of the COVID-19 pandemic were also included in the evaluation. The first lockdown of the COVID-19 pandemic started in Germany on March 22, 2020. From this point on, almost no F2FGs were allowed to occur. To compare the different utilization patterns, we divided the participants into 2 groups. The pre–COVID-19 group consisted of patients whose T0 occurred before November 22, 2019. These patients had at least 4 months to attend an F2FG before lockdown. The COVID-19 group included patients whose study period was predominantly during the COVID-19 lockdown.

### Ethics Approval and Study Registration

The ethics committee of the medical faculty at the Technical University Dresden approved the study protocol (EK 350082016). Approval from 19 additional ethics committees was obtained from the associations of the physicians at the federal state level and from the participating university hospitals. We registered the underlying RCT within the German Clinical Trials Register (DRKS-ID DRKS00014627) [18]. Before inclusion in the study, all patients received written study information. Oral and written informed consent was obtained during the medical consultation. The pseudonymized study data were collected involving an independent data custodian and in compliance with all data protection regulations.

### Statistical Analyses

We used descriptive statistics for the sociodemographic and clinical characteristics of the study population. For group comparisons, we used the chi-square or McNemar test for nominal variables and the *t* test for ordinally scaled data (2-sided, α=.05). All calculations were performed with SPSS (version 28.0; IBM Corp).

## Results

### Study Size

**Figure 1:**
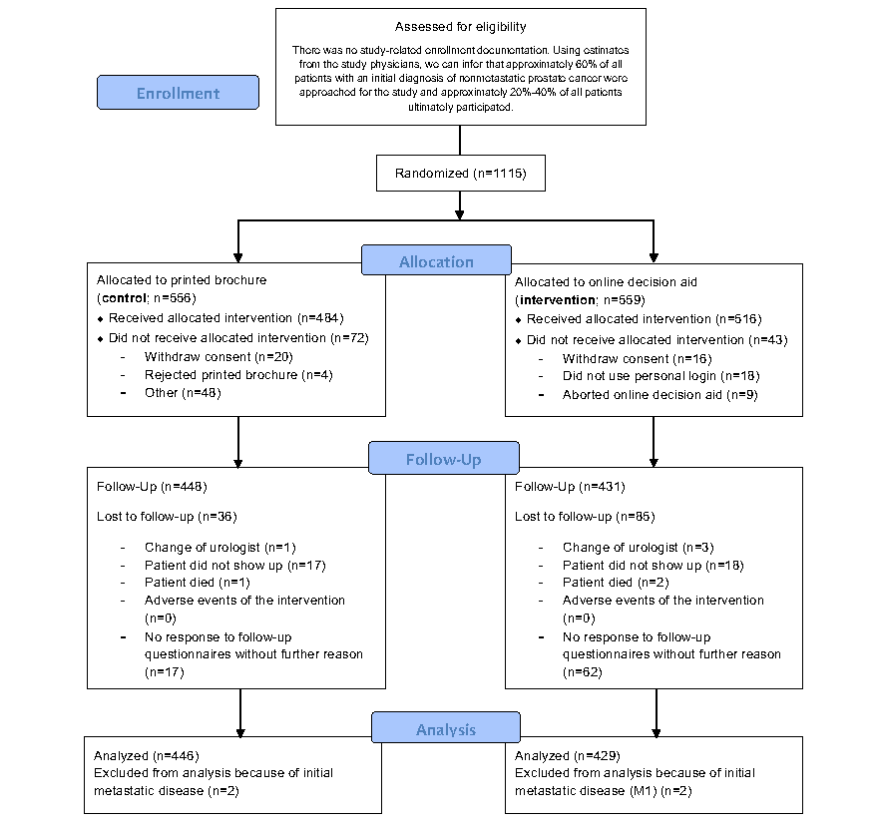
CONSORT (Consolidated Standards of Reporting Trials) flow diagram of the underlying randomized controlled trial. EvEnt-PCA: Evaluation of a patient-oriented decision aid and the German health care situation in nonmetastatic prostate cancer.

Figure 1 shows the CONSORT (Consolidated Standards of Reporting Trials) flow diagram of this underlying RCT (Multimedia Appendix 2) [19]. The average time period between T0 and T1 was 13.4 months (SD 2.9 months; range 6.1-34 months). The questionnaires were completed by 1000 patients at T0 and 871 patients at T1. After excluding missing data, our results were based on the responses of 687 patients and 116 urologists with complete datasets at T0 and T1. Among the patients, 308 (44.8%) were in the pre–COVID-19 group and 379 (55.2%) were in the COVID-19 group. The patients had a mean age of 67.1 (SD 6.8; range 44-81) years; 594 (89.2%) were in a partnership, 567 (85.1%) were parents, 608 (91.2%) spoke German as their native language, and 455 (68.2%) lived in towns with more than 10,000 inhabitants. OSG users reported less income or did not provide this information more frequently compared to nonusers. Beyond this, there were no differences (Table 1). Physicians had a mean age of 48.3 (SD 8.2) years, and they had a work experience of 14.7 (SD 7.8) years; 96 (92.3%) were males and 100 (96.2%) worked in places with more than 10,000 inhabitants. The majority (75/104, 72.1%) worked together with other urologists (Table 2).

**Table 1:** Participants’ baseline data and comparison of online support group nonusers versus online support group users.

		All participants (N=687)	Online support group nonusers	Online support group users	*P* value (*χ*² test)
Age (years) (OSG^a^ nonusers=521; OSG users=165), mean (SD)		67.1 (6.8)	67.3 (6.9)	66.5 (6.7)	.18
**Family status (OSG nonusers=508; OSG users=158), n (%)**					
	Single (eg, divorced, widowed)	69 (10.4)	52 (10.2)	17 (10.8)	N/A^b^
	Married or in permanent partnership	594 (89.2)	455 (89.6)	139 (88)	.21
	Other	3 (0.5)	1 (0.2)	2 (1.3)	N/A
**Children (OSG nonusers=509; OSG users=157), n (%)**					
	Yes	567 (85.1)	428 (84.1)	139 (88.5)	.17
	No	99 (14.9)	81 (15.9)	18 (11.5)	N/A
**Education (OSG nonusers=509; OSG users=160), n (%)**					
	A-level	277 (41.4)	207 (40.7)	70 (43.8)	N/A
	Middle	202 (30.2)	161 (31.6)	41 (25.6))	.56
	Low	141 (21.1)	107 (21)	34 (21.3)	N/A
	Other	49 (7.3)	34 (6.7)	15 (9.4)	N/A
**Income (OSG nonusers=505; OSG users=161), n (%)**					
	<US $1600	55 (8.3)	46 (9.1)	9 (5.6)	N/A
	US $1600-4300	417 (62.6)	306 (60.6)	111 (68.9)	.02
	> US $4300	138 (20.7)	116 (23)	22 (13.7)	N/A
	Not specified	56 (8.4)	37 (7.3)	19 (11.8)	N/A
**Place of residence (OSG nonusers=508; OSG users=159), n (%)**					
	<10,000 inhabitants	212 (31.8)	153 (30.1)	59 (37.1)	.10
	>10,000 inhabitants	455 (68.2)	355 (69.9)	100 (62.9)	N/A
**Gleason score^c^** **(OSG nonusers=522; OSG users=165), n (%)**					
	6	33 (4.8)	23 (4.4)	10 (6.1)	N/A
	7	310 (45.1)	232 (44.4)	78 (47.3)	N/A
	8	32 (4.7)	23 (4.4)	9 (5.5)	.62
	9	39 (5.7)	33 (6.3)	6 (3.6)	N/A
	10	0 (0)	0 (0)	0 (0)	N/A
	Unknown	273 (39.7)	211 (40.4)	62 (37.6)	N/A
**cT-stadium^d^** **(OSG nonusers=522; OSG users=165), n (%)**					
	T2a	24 (3.3)	19 (3.6)	5 (3)	N/A
	T2b	8 (1.2)	8 (1.5)	0 (0)	N/A
	T2c	239 (34.8)	183 (35.1)	56 (33.9)	.50
	T3a	92 (13.4)	70 (13.4)	22 (13.3)	N/A
	T3b	57 (8.3)	39 (7.5)	18 (10.9)	N/A
	Unknown	8 (1.2)	5 (1)	3 (1.8)	N/A
**cN-stadium^d^** **(OSG nonusers=522; OSG users=165), n (%)**					
	N0/x	566 (82.4)	431 (82.6)	135 (82)	N/A
	N1	15 (2.2)	12 (2.3)	3 (2)	.95
	Unknown	106 (15.4)	79 (15.1)	27 (16)	N/A
**Randomized controlled trial group allocation (OSG nonusers=522; OSG users=165), n (%)**					
	Intervention	330 (48)	241 (46.2)	89 (53.9)	N/A
	Control	357 (52)	281 (53.8)	76 (46.1)	.08

^a^OSG: online support group.

^b^N/A: not applicable.

^c^The Gleason score is a grading system for prostate cancer aggressiveness ranging from well-differentiated Gleason 6 to highly aggressive Gleason 10.

^d^Tumor characteristics are classified according to the current TNM (tumor, node, metastasis) classification for prostate cancer [20]. The multidimensional classification indicates different degrees of severity with regard to the extent (size, infiltration depth) of the primary tumor (T), lymph node involvement (N), and the occurrence of metastases (M). A preceding c means that the determination was made by a clinical examination.

**Table 2:** Characteristics of the urologist respondents (n=104; no data available for 12 urologists).

			Values
Age (years), mean (SD)			48.3 (8.2)
Duration of profession (years), mean (SD)			14.7 (7.8)
**Sex, n (%)**			
	Male	96 (92.3)	
	Female	8 (7.7)	
**Place of work, n (%)**			
	<10,000 inhabitants	4 (3.8)	
	>10,000 inhabitants	100 (96.2)	
**Urological workplace (multiple options possible), n (%)**			
	Single	29 (27.9)	
	Together with other urologists	45 (43.3)	
	Urological clinic	36 (34.6)	
	Other	8 (7.7)	

### Utilization of Self-Help

Only 7 patients attended an F2FG, and all of them were from the pre–COVID-19 group. Thus, no patient in the COVID-19 group attended an F2FG (7/308, 2.3% vs 0/379, 0%, respectively; *P*=.003). Most patients (471/687, 68.6%) reported having no interest in F2FGs. Another 122 (17.8%) of the 687 patients did not know about F2FGs, and 66 (9.6%) had other reasons for not attending. OSGs were used by 165 (24%) patients. The frequency of OSG use did not change significantly before (76/308, 24.7%) or during (89/379, 23.5%) the COVID-19 pandemic (*P*=.72). Only 3 (1.8%) OSG users actively posted messages. Of the 7 users of F2FGs, 4 (57%) also used OSGs. Due to the minimal F2FG usage, we focused on OSG users versus OSG nonusers (Table 3).

Of those who participated in OSGs, 3 (1.8%) actively participated, and 46 (27.9%) reported that OSG was helpful or slightly helpful for decision-making. A similar number of respondents thought it was not helpful (47/121, 38.8%). Although 19 (4%) nonusers regretted their treatment decisions, only 1 (0.7%) OSG user did (*P*=.03). More often, OSG users rather than nonusers reported that OSGs were mentioned by their physicians (*P*<.001).

**Table 3:** Comparison of online support group nonusers and online support group users.

			Online support group nonusers (n=522), n (%)		Online support group users (n=165), n (%)		*P* value (*χ*² test)	
Active role in online support groups			N/A^a^		3 (1.8)		N/A	
**Usefulness of online support groups for treatment decisions (n=121)**								N/A
	Helpful	N/A		11 (9.1)				
	Slightly helpful	N/A		35 (28.9)				
	Minimally helpful	N/A		28 (23.1)				
	Not helpful	N/A		47 (38.8)				
Online support groups changed treatment decisions			N/A		5 (4.1)			
**Regret of treatment decisions**								.03
	Strongly agree	19 (4)		1 (0.7)				
	Neutral	15 (3.1)		10 (6.5)				
	Strongly disagree	443 (92.9)		142 (92.8)				
Physician mentioned online support groups			31 (5.9)		26 (15.7)		<.001	

^a^N/A: not applicable.

### Consistency of Statements From Patients and Physicians

Table 4 shows the comparisons between the physicians’ and patients’ statements about whether F2FGs and OSGs were mentioned in their conversations. In most cases, patients and physicians agreed that F2FGs and OSGs were not mentioned in their conversations. In 140 (20.4%) of the 686 conversations, patients and physicians disagreed that F2FGs were mentioned, and in 94 (13.7%) conversations, there was disagreement regarding OSGs.

Table 5 shows the number of patients who visited F2FGs and OSGs and how often doctors assumed this. Only 1 out of 7 patients (14%) who attended an F2FG and 5 out of 165 (3%) patients who attended an OSG were correctly assessed by the physicians. Physicians rated the use of F2FGs at 1.5% (10/687) and OSG at 2.6% (18/687), while in fact, F2FGs were visited by 1% (7/687) and OSGs by 24% (165/687) of the patients (*P*=.61 and *P*<.001, respectively).

**Table 4:** Comparison of physician and patient statements about whether peer-to-peer support groups were mentioned in their conversations.

	Face-to-face support group mentioned (n=686), n (%)	Online support group mentioned (n=686), n (%)
Both agreed: not mentioned	528 (77)	586 (85.4)
Both agreed: mentioned	18 (2.6)	6 (0.9)
Disagree: patients yes, physicians no	69 (10.1)	51 (7.4)
Disagree: patients no, physicians yes	71 (10.3)	43 (6.3)

**Table 5:** Comparison of physician and patient statements about whether face-to-face groups and online support groups were accessed.

	Face-to-face support group visited (n=687), n (%)	Online support group visited (n=687), n (%)
Both agreed: not visited	670 (97.5)	508 (73.9)
Both agreed: visited	1 (0.1)	5 (0.7)
Disagree: patients yes, physicians no	6 (0.9)	160 (23.3)
Disagree: patients no, physicians yes	9 (1.3)	13 (1.9)

### Impact on Distress, Depression, and Anxiety

Table 6 presents the results of the screening questionnaires for distress, depression, and anxiety (Patient Health Questionnaire-4 items). At both time points, scores for depression and anxiety among nonusers were lower than those among users (all *P*<.05), while distress showed the same trend (*P*=.06 and *P*=.07, respectively). Comparing psychological burden at baseline (T0) versus follow-up (T1) (Table 7), it improved for distress and anxiety in all subgroups (*P*=.003), while depression showed the same trend in the nonuser group (*P*=.06 and *P*=.66, respectively).

**Table 6:** Results for distress, depression, and anxiety after initial diagnosis (T0) and at the follow-up of 1 year (T1) (comparison of online support group nonusers and online support group users).

		Online support group nonusers (n=522), mean (SD)	Online support group users (n=165), mean (SD)	*P* value (*t* test comparing nonusers and users)
Distress^a^		4.5 (2.8)	5.0 (2.6)	.07
**T0 (after initial diagnosis)**				
	Depression	0.9 (1.2)	1.2 (1.3)	.03
	Anxiety	1.0 (1.3)	1.3 (1.3)	.02
	Distress	3.1 (2.4)	3.5 (2.3)	.06
**T1 (at a follow-up of 1 year)**				
	Depression	0.8 (1.1)	1.1 (1.2)	.003
	Anxiety	0.7 (1)	1.2 (1.2)	.001

^a^The Distress Thermometer contains the values 0 (no) to 10 (maximum distress). Values >4 are considered conspicuous.

**Table 7:** Results for distress, depression, and anxiety after initial diagnosis (T0) and at the follow-up of 1 year (T1) (comparison of changes over time).

	Online support group users (*P* value)	Online support group nonusers (*P* value)
Distress	<.001	<.001
Depression	.06	.66
Anxiety	<.001	.003

## Discussion

In our study, we observed that patients with newly diagnosed nonmetastatic PC in Germany rarely engaged with F2FGs (7/687, 1%), while the use of OSGs was more common (165/687, 24%). The majority of the treating physicians were unaware of this fact, as they massively underestimated OSG use (18/687, 2.6% estimated use vs 165/687, 24% actual use). Patients who used OSGs had significantly higher scores for depression and anxiety both before and after OSG attendance.

### Decrease in F2FG Use

F2FGs were more often mentioned by urologists, but they played only a minor role for patients with newly diagnosed nonmetastatic PC. In the pre–COVID-19 group, only 2.3% (7/308) of the patients attended F2FGs. Approximately 1 decade earlier, this percentage was 7.6% among patients with different cancers and disease stages or even higher [21,22]. The decline in local F2FG use can also be found on the website of the German Federal Association of Prostate Cancer Support Groups (BPS). Approximately 10 years ago, during one of our surveys, 230 local groups existed [5]; in 2023, 180 (78%) of these groups were still listed [23]. The COVID-19 pandemic lockdown paused F2FG activities almost completely. In our work, we analyzed OSG use before and during the COVID pandemic. In contrast to other studies, wherein differences were found in the frequency and format before and during the COVID-19 pandemic, we have no evidence that COVID-19 pandemic has acted as a catalyst for the use of web-based media [24]. OSGs were used by 165 (24%) of the 687 patients. The frequency of OSG use did not change significantly before (76/308, 24.7%) and during (89/379, 23.5%) the pandemic (*P*=.72). Overall, F2FGs completely disappeared during the pandemic and did not even play a pronounced role in this disease phase before.

### Growing Needs for Moderated OSGs

It seems necessary for peer-to-peer support groups to adapt to structural changes [25,26]. In addition to the established F2FGs, the need for moderated OSGs is increasing [27-29]. Anonymous use and constant availability facilitate participation in OSGs, meaning that a high proportion of patients make use of these services. This fact is not recognized by treating physicians. It may even be possible in the future to realize some of the unique selling points of F2FGs online. A key issue could consist of dividing OSGs according to specialized topics and restricting group sizes. Training F2FG instructors and their experience with group dynamics could provide a good basis for optimizing OSGs by mediating social exchange in small groups. However, dealing with modern communication media and changing ways of meeting are still challenges for older patients [22]. At the same time, there will always be certain content that remains dependent on presence such as excursions and social events. Anonymity can also be seen as a disadvantage here.

According to our clinical judgment, F2FGs do not play a relevant role in this early disease phase of patients with nonmetastatic PC considering curative treatment. The majority of these patients initially come to terms with the disease after their treatment. The significance of F2FGs might change for individual patients in the event of disease recurrence or systemic disease. These developments are typically the beginning of a longer disease phase without a curative treatment approach and with a greater psychological burden. Due to the longer disease period of years or even decades, regional F2FGs are then suitable for building sustainable personal relationships and friendships. This social integration might support the coping process [5].

### Misconceptions of Physicians

Although the limited use of F2FGs was correctly estimated by the treating physicians, the use of OSGs was underestimated. Knowing and accepting OSGs as frequent sources of information might benefit patient-physician communication. Some physicians’ problems concerning their patients’ needs and perceptions, such as health beliefs or information needs, are already known [30,31]. Our results demonstrate that this problem also concerns physicians’ perceptions of their patients' need for OSGs as well as their actual attendance of OSGs. However, communication about these issues seems to be rather limited in conversations held with physicians.

### Patient Characteristics of Peer-To-Peer Support Group Attendees

OSGs are particularly suitable for young patients or for individuals with rare tumors for which local self-help groups rarely exist due to too few patients [32-34]. Approximately one-third of our study patients who attended peer-to-peer support services rated them helpful. On the other hand, this approach rarely affected the final treatment decision. This result is contrary to our earlier findings (4% vs 29%) [5]. The reason may be that there was a smaller sample of patients using peer-to-peer support in our study, because the underlying RCT recruited patients from routine care. Our previous work recruited users of F2FGs and OSGs and was therefore prone to selection bias. In this respect, our results represent a credible order of magnitude for German routine care. Participants who used peer-to-peer support services had higher scores for anxiety and depression in our study. Patients with greater emotional stress are naturally more likely to seek support. In this respect, these results appear plausible. In another study, a more active emotion-oriented coping style significantly predicted F2FG participation [21]. Due to the overall minor effects, these authors concluded that peer-to-peer support group participation was not related mainly to psychosocial distress. Anxiety and distress decreased significantly after 1 year. However, this effect also occurred for nonusers, so that these changes are probably due to the known course after local treatment [35].

### Limitations

One principal limitation of our survey is that we did not ask any specific details about the OSG, as the questionnaire was already very extensive. The question of the content or the effects of different forms of OSGs are what we asked in earlier studies, where we showed that OSGs play a significant role in patients' treatment decision-making and for the social environment of patients with PC [10,36,37]. Information exchange in OSGs was predominant, but emotional and supportive content also had an important function. Due to the large scope of the primary study, reductions had to be made such as the difficulties in retaining users in the long term, as opposed to initially attracting them [38,39]. In this study, we focused on whether self-help services were used at all, and if so, for how long. We also asked how helpful the self-help program was in the treatment decision and whether this had changed the treatment decision. However, in Germany, there is a dominant OSG organized by the BPS [4,5,10]. The BPS forum is the largest offering in the German-speaking countries. We strongly assume that a majority of patients have used this BPS forum, but we cannot tell for sure.

In the context of our study, the 1-year period after diagnosis was surveyed. It was assumed that several patients used peer-to-peer support services for the first time after this period. Therefore, our percentages cannot be interpreted as final user ratios of peer-to-peer support. Moreover, our study is a predefined secondary analysis of an RCT. The available study sample was determined by the initial sample size calculation and the questionnaire response rate. The sample obtained was large but methodologically only suitable for exploratory analyses. Due to the context of an extensive questionnaire survey, there are possible limitations in terms of response validity. Both the doctors and the patients were blinded to each other’s answers. This makes relevant social desirability bias very unlikely.

The strengths of our study include the very moderate patient selection due to motivated recruiting centers and the large sample size from routine care. This means that besides the study intervention consisting of different modes of patient information, all participants were treated under the regular circumstances of the German health care system. As the items concerning peer-to-peer support utilization were secondary aspects within the RCT, possible bias might have been reduced. The generalizability of the findings to other health care systems and societies appears limited. Moreover, the results are not generalizable to other phases of the disease, other entities, or other genders. However, there is a larger knowledge gap for male patients than for those with well-researched gynecological entities such as breast cancer.

### Conclusions

Traditional F2FGs are mentioned more often by urologists than OSGs are, but even before the COVID-19 pandemic, they played only a minor role for patients with newly diagnosed nonmetastatic PC. Our study shows that OSGs are much more relevant and frequently used. One out of 4 patients uses OSGs, and one-third of these patients consider them helpful for treatment decision-making. Nevertheless, in contrast to former evidence, this approach rarely affects the final treatment decision. Physicians significantly underestimate the frequency with which OSGs are used by their patients. Peer-to-peer support groups are more likely to be attended by patients with elevated levels of anxiety and depression. Although our study provides valuable information on the current use of peer-to-peer support groups, future research should focus on its effects. Comparative interventional trials are needed to recommend peer-to-peer interventions in a more targeted way and to better identify patients’ needs.

## Appendix

Multimedia Appendix 1 EvEnt-PCA (Evaluation of a patient-oriented decision aid and the German health care situation in nonmetastatic prostate cancer) case report form questionnaire: questions related to self-help.

Multimedia Appendix 2 CONSORT-eHEALTH checklist (V 1.6.1).

## Abbreviations

BPS: Bundesverband Prostatakrebs Selbsthilfe e.V./Federal Association of Prostate Cancer Support Groups
CONSORT: Consolidated Standards of Reporting Trials
F2FG: face-to-face support group
OSG: online support group
PC: prostate cancer
RCT: randomized controlled trial

## Data Availability

This was a secondary analysis of data from a randomized controlled trial comparing an online decision aid versus a printed brochure for patients with nonmetastatic prostate cancer [11]. Data are also deposited in publicly available repositories via the result report on the homepage of the German Innovation Fund of the Federal Joint Committee and on request to the authors of the study [40]. We disclose that generative artificial intelligence was not used in any part of the manuscript.
