# Supplementary material for: Urologists’ Estimation of Online Support Group Utilization Behavior of Their Patients With Newly Diagnosed Nonmetastatic Prostate Cancer in Germany: Predefined Secondary Analysis of a Randomized Controlled Trial
Source: J Med Internet Res. 2025 Apr 7;27:e56092. doi: 10.2196/56092 (PMC12012397; doi:10.2196/56092)
Supplement: Multimedia Appendix 1 [file jmir_v27i1e56092_app1.pdf]

Supplement

EvEnt-PCA CRF Dokumentationsbögen: Fragen mit Bezug zu Selbsthilfe/ EvEnt-PCA CRF Questionnaire: Questions related to self-help

Arzt Stammdaten (T0)/Physician master date (T0)

Zur Beratung bei neu diagnostiziertem nicht metastasierten Prostatakarzinom nutze ich neben dem Gespräch...(Mehrfachantwort)/For counselling in newly diagnosed non-metastatic prostate cancer, I use the following in addition to the consultation...(multiple answers)

- ☐ keine weitere Unterstützung/No further support.
- ☐ Printmedien/Printed media.
- ☐ Videos.
- ☐ Internetquellen/Internet sources.
- ☐ Entscheidungshilfe für Patienten/Decision aid for patients.
- ☐ Kontaktangebot zur Selbsthilfe/Contact to self-help group.

Arzt Abschlussdokumentation (T2)/Physician final documentation (T2)

Unterstützungsangebot/Offer of support

Sind in den Gesprächen mit Ihrem Patienten seit Diagnosestellung der Prostatakarzinom-Erkrankung folgende Unterstützungsangebote zur Sprache gekommen? (Mehrfachantwort)/Have the following support services been discussed with your patient since the diagnosis of prostate cancer? (multiple answers)

- ☐ Rehabilitationsmaßnahme/Rehabilitation program
- ☐ Psychologe oder Psychoonkologe/Psychologist or psycho-oncologist
- ☐ Sozialarbeiter/Social worker
- ☐ Klassische Selbsthilfe (Selbsthilfegruppe)/Traditional self-help (self-help group)
- ☐ Online-Selbsthilfe (Internetforum)/Online self-help (internet forum)
- ☐ Andere/Other
- ☐ Keine der aufgeführten Angebote/None of the services listed

Hat Ihr Patient seit Diagnosestellung der Prostatakarzinom-Erkrankung folgende Unterstützung in Anspruch genommen? (Mehrfachantwort)/Has your patient received the following support since being diagnosed with prostate cancer? (multiple answers)

- ☐ Rehabilitationsmaßnahme/Rehabilitation program
- ☐ Psychologe oder Psychoonkologe/Psychologist or psycho-oncologist
- ☐ Sozialarbeiter/Social worker
- ☐ Klassische Selbsthilfe (Selbsthilfegruppe)/Traditional self-help (self-help group)
- ☐ Online-Selbsthilfe (Internetforum)/Online self-help (internet forum)
- ☐ Andere/Other
- ☐ Keine der aufgeführten Angebote/None of the services listed

Patient Abschlussdokumentation (T2)/Patient final documentation (T2)

Sind in den Gesprächen mit Ihrem Arzt seit Feststellung der Prostatakrebs-Erkrankung folgende Unterstützungsangebote zur Sprache gekommen? (Mehrfachantwort)/Have the following support services been discussed with your doctor since you were diagnosed with prostate cancer? (multiple answers)

- ☐ Rehabilitationsmaßnahme („Kur“)/Rehabilitation program
- ☐ Psychologe oder Psychoonkologe/Psychologist or psycho-oncologist
- ☐ Sozialarbeiter/Social worker
- ☐ Selbsthilfegruppe (persönliches Treffen)/Self-help group (face-to-face meeting)
- ☐ Online-Selbsthilfe (Internetforum)/Online self-help (internet forum)
- ☐ Andere/Other
- ☐ Keines der aufgeführten Angebote/None of the services listed

Haben Sie seit Bekanntwerden Ihrer Prostatakrebs-Erkrankung folgende Unterstützung in Anspruch genommen?/ Have you taken advantage of the following support since you became aware of your prostate cancer?

- ☐ Rehabilitationsmaßnahme („Kur“)/rehabilitation program
- ☐ Psychologe oder Psychoonkologe/Psychologist or psycho-oncologist
- ☐ Sozialarbeiter/Social worker
- ☐ Selbsthilfegruppe (persönliches Treffen)/Self-help group (face-to-face meeting)
- ☐ Online-Selbsthilfe (Internetforum)/Online self-help (internet forum)
- ☐ Andere/Other
- ☐ Keines der aufgeführten Angebote/None of the services listed

Nutzung von Selbsthilfeangeboten/Utilisation of self-help services

Haben Sie seit Feststellung Ihrer Prostatakrebs-Erkrankung Angebote der Selbsthilfe genutzt? Haben Sie im Internet schon einmal ein Forum für Betroffene mit Prostatakrebs besucht?/Have you used self-help services since you were diagnosed with prostate cancer? Have you ever visited a forum on the Internet for people affected by prostate cancer?

- ☐ Nein, da ich kein Interesse daran habe/No, because I am not interested.
- ☐ Nein, da ich dieses Angebot nicht kenne/No, because I am not familiar with this offer.
- ☐ Ja, ich habe bis zu 1 Stunde darin gelesen/Yes, I have read it for up to 1 hour.
- ☐ Ja, ich habe länger als 1 Stunde darin gelesen/Yes, I have read it for more than 1 hour.
- ☐ Ja, ich habe dort auch schon selbst geschrieben (Frage oder Beitrag)/Yes, I have already written there myself (question or contribution).

Waren Sie schon einmal auf dem Treffen einer Prostatakrebs-Selbsthilfegruppe?/Have you ever been to a prostate cancer self-help group meeting?

- ☐ Nein, da ich kein Interesse daran habe/No, because I am not interested.
- ☐ Nein, da ich dieses Angebot nicht kenne/No, because I am not familiar with this offer.
- ☐ Nein, aus anderen Gründen (Zeitmangel, Entfernung, usw.)/No, for other reasons (lack of time, distance, etc.).
- ☐ Ja, ich war 1- bis 2-mal dort/Yes, I have been there 1 or 2 times.
- ☐ Ja, ich war häufiger als 2-mal dort/Yes, I have been there more than 2 times.
- ☐ Ja, ich bin aktives Mitglied/Yes, I am an active member.

Wie hilfreich war das Selbsthilfeangebot auf Ihrem Weg zu einer Behandlungsentscheidung?/ How helpful was the self-help program on your way to deciding on treatment?

- ☐ Überhaupt nicht/Not at all
- ☐ Wenig/Little
- ☐ Etwas/Somewhat
- ☐ Sehr/Very

Hat sich durch das Selbsthilfeangebot Ihre Behandlungsentscheidung verändert?/Has the self-help program changed your treatment decision?

- ☐ Ja/Yes
- ☐ Nein/No
- ☐ Keine Angabe/Not specified
